# A methodology for establishing historical wetland habitat change in Irish freshwater pearl mussel catchments

**DOI:** 10.1007/s10661-025-14206-z

**Published:** 2025-06-27

**Authors:** Barry G. Walls, Evelyn A. Moorkens, Jeremy J. Piggott

**Affiliations:** School of Natural Sciences, Discipline of Zoology, Trinity College Dublin, The University of Dublin, Dublin, Ireland

**Keywords:** Freshwater Pearl Mussel, Hydrology, Wetland, Catchment restoration, Nature Restoration Law, Ecosystem services

## Abstract

Since the 1700s, global wetlands have declined by 3.4 million km^2^. Wetland quality loss is a key driver of freshwater pearl mussel (FPM) population decline in peaty catchments. GIS techniques were used to determine wetland cover in 1834 and 2023, in eight peaty FPM catchments in Ireland, based on historical Ordnance Survey mapping (1834) and Irish National Land Cover (NLC) mapping (2023). Historical catchment wetland change (1834–2023) ranged between a net loss of 7.03 to 29.96%. Wetland coverage in 2023 varied between 53.85 and 83.53%. In 1834, that coverage ranged from 59.72 to 89.85%. The Hydromusindex (HDi) was developed to evaluate catchment-scaled historical wetland change, which for each of the catchments studied was a net loss. This damage index was based on the ratio of the 2023 catchment wetland coverage in proportion to the 1834 baseline scenario, and the catchment proportional coverage of each wetland cover type weighted by their potential contribution towards water storage; higher HDi values indicate increased damage. The HDi values of eight studied catchments ranged from 21 to 46. The HDi can be used to assess catchment restoration design and to rank FPM catchments for restoration, under the Nature Restoration Law. Measurable targets for catchment-scaled restoration have been produced. The sum of weighted wetland scores, in contrast to predefined reference values, can be used for rapid damage evaluation. An estimated 41.06% of Ireland’s plantation forestry is located on peatlands and peat soils, thereby highlighting considerable opportunities in terms of meeting the EU Nature Restoration Law’s targets.

## Introduction

Global wetlands have declined by 3.4 million km^2^ since 1700, with the net loss approaching 21% (Fluet-Chouinard et al., 2023). Peatland accounts for at least 50% of the world’s terrestrial wetland ecosystems, providing critical functions such as biodiversity support and water regulation (Habib et al., 2024). Up to 50% of global wetlands have been drained in the last century (Davidson, 2014), with more than half of Europe’s peatlands lost during the same period (Andersen et al., 2017). Nearly 90% of Ireland’s peatlands are now degraded (Fluet-Chouinard et al., 2023). In 1981, Blanket Bog covered 897,556 ha (approximately 13%) of Ireland, but that has been disproportionally targeted for state-funded monoculture forestry, consisting primarily of non-native evergreen species (Flynn et al., 2022; Hammond, 1981), with consequential impacts on freshwater habitats and species.

The holarctic species freshwater pearl mussel *Margaritifera margaritifera* (L. 1758) remains globally ‘Endangered’ (Moorkens et al., 2017) and Critically Endangered within Europe (Moorkens, 2024). This endangered mollusc is listed in Annex II and V of the EU Habitats Directive (92/43/EEC) (Council of the European Union, 1992).

Between 1920 and 2010, *M. margaritifera* declined by an estimated 81.5% within its European range (Moorkens, 2011). Almost 90% of Europe’s *M. margaritifera* population was lost during the twentieth century (Bauer, 1988; Cuttelod et al., 2011). Ireland’s population was estimated at 12 million individuals in 2009, accounting for almost 46% of the European Union population (Moorkens et al., 2017). Up to 32.7% has since been lost between the last two Article 17 reporting periods (i.e. 2007–2012 and 2013–2018) alone (National Parks & Wildlife Service, 2019). A reduced national population of 600,000 individuals is forecast towards 2100, based on an observed decline of c. 96% over the previous three generations (Moorkens et al., 2017).

Many European freshwater pearl mussel populations are approaching ecological marginality, and the risk of both functional and species extinction has never been higher (Lopes-Lima et al., 2017; Tamario et al., 2022). Several European populations have already succumbed to functional extinction within a human lifetime (Geist & Kuehn, 2005; Geist et al., 2018; Lopes-Lima et al., 2017). Successful recruitment is now a rarity, even in Europe’s priority rivers (IUCN, in press). Juvenile mussels are buried in riverbed gravels, and this substrate must remain clean for at least 5 years until the mussels are large enough to move to the surface and withstand flowing water. Both physical and organic-driven sedimentation can cut off the oxygen supply to juvenile mussels, leading to death (Moorkens, 2020). Sufficient near-bed velocity is needed to keep substrate gravels clean, and this velocity is driven by catchment hydrology conditions (Moorkens & Killeen, 2014).

Extinction of many European freshwater pearl mussel populations is expected within a few generations, driven by a legacy of overlapping issues including wetland loss, agricultural intensification, forestry, water abstraction, development, and Member States’ failure to adequately meet the requirements of the 1992 EU Habitats Directive (IUCN, in press). Within recent decades, sizeable mussel mortality events have occurred in some of Ireland’s premiere *Margaritifera* catchments during low flow conditions, where large mussel beds have become exposed. These losses are attributed to hydrological effects associated with historical wetland loss and damage, land management practices, forestry, and water abstraction and flow regulation. Out of 46 freshwater pearl mussel captive-breeding conservation projects throughout 16 European countries, none have achieved long-term significant improvement (Geist et al., 2023). Catchment restoration is considered to be the most essential effort needed to reverse the decline of the species (Geist, 2010; Moorkens, 2010; IUCN, in press).

Wetlands, lakes and groundwater are the principal mechanisms of hydrological storage in peaty catchments (Acreman & Bullock, 2003; Goodbrand et al., 2019). Wetlands and lakes attenuate floods and augment base flows (McLaughlin & Cohen, 2014; Nepal et al., 2024). The restoration of catchment hydrological functioning is integral for the provision of adequate flow regimes in peaty *Margaritifera* catchments (Farrell et al., 2024; Rasmussen et al., 2018). Hopes focus on the EU’s Nature Restoration Law to drive *M. margaritifera* conservation and prevent forecasted extinction. Article 4 of The Nature Restoration Law (EU 2024/1991) outlines that EU Member States are required to restore at least 30% of the total area of all Annex I habitats that are not in *good* condition by 2030, increasing to 60% by 2040, and 90% by 2050 (Mäkipää et al., 2024). However, the lack of defined restoration targets for catchment-scaled restoration of hydrological functioning restricts progress (Arthington et al., 2018). Furthermore, a data gap exists regarding the development of a modelling method suitable for evaluating the effects of relevant stressors on *Margaritifera* populations.

Several indices have been developed for modelling large spatial-scaled wetland loss and degradation, and for wetland management purposes. Mao et al. (2018) and Lv et al. (2019) employed remote sensing indices for large-scale analysis relating to wetland conversion to farmland and wetland loss in China. Wang et al. (2020) analysed Landsat imagery using the Google Earth Engine (GEE) to evaluate annual changes in coastal tidal flats in China between 1986 and 2016. A Wetland Damage Index (WDI) was published in 2022 by Huang et al. (2022) for evaluating wetland destruction and risk in China, based on wetland loss between 2000 and 2020, environmental stress caused by human activities, and remote sensing-based ecological indices. Ridwan et al. (2024) developed a land degradation index (LDI) by combining indices from multiple remote-sensing sources for use in monitoring wetland degradation in Indonesia. Each damage index has a unique application, depending on individual project goals and aims. To our knowledge, a damage index for evaluating historical wetland loss and the remaining coverage of essential peatland habitat mosaics (i.e. peatlands and heaths) in peaty freshwater pearl mussel catchments has not yet been developed, thereby limiting progress in catchment restoration design. We aimed to develop (a) a damage index (Hydromusindex) that evaluates historical wetland loss and (b) produce measurable data-based targets for catchment-scaled restoration design.

The objectives of this study are as follows:

1. Select eight peaty freshwater pearl mussel sample catchments for analysis, which include priority Special Area of Conservation (SACs) and catchments associated with modification, based on current literature (Moorkens, 2010).
2. Conduct a detailed investigation of the wetland cover and wetland loss within the sample catchments between 1834 and 2023, based on GIS analysis of the first series Ordnance Survey of Ireland (OSI) mapping (1834) and Ireland’s National Land Cover Map (2023) dataset shapefiles. We define historical wetland loss as the net decline in functional wetlands between the periods, including wetland habitats that are either destroyed or damaged but that can be either partially or fully hydrologically restored.
3. Develop a ‘damage index’ that characterises and evaluates historical wetland loss within the catchments.
4. Establish measurable data-based targets for the restoration of wetlands in freshwater pearl mussel catchments.
5. Investigate the current forestry coverage within the sample catchments to determine opportunities for wetland restoration.

## Materials and methods

### Study area

**Fig. 1:**
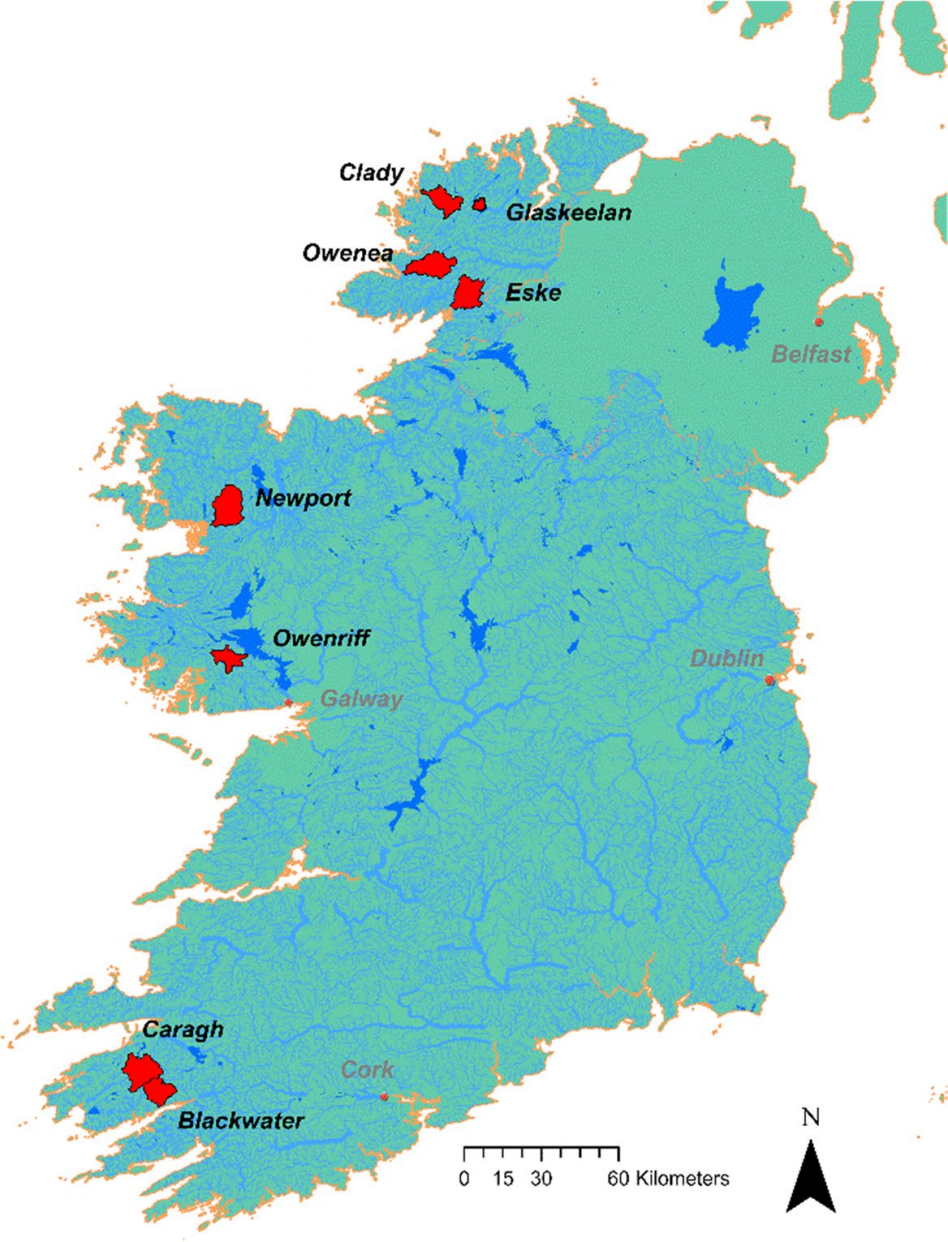
Locations of the studied catchments

Eight freshwater pearl mussel ‘peaty’ catchments in the Republic of Ireland were selected for analysis, based on a review of historical monitoring data (Moorkens, 2010) (Table 1). Four sample sites (no. 1–4) are listed within Ireland’s ‘Top 8’ freshwater pearl mussel catchments, in terms of population size and recruitment status (Moorkens, 2010), and are thus considered to be relatively less modified (hereafter referred to as ‘less modified’ catchments) (Fig. 1).

**Table 1:** Sample catchments

No	Catchment	Country	Area (km^2^)
1	Glaskeelan	Co. Donegal	17.45
2	Owenriff	Co. Galway	67.41
3	Blackwater	Co. Kerry	88.27
4	Caragh	Co. Kerry	133.61
5	Clady	Co. Donegal	89.04
6	Eske	Co. Donegal	113.90
7	Owenea	Co. Donegal	126.09
8	Newport	Co. Mayo	146.49

### Mapping

Table 2 outlines the mapping sources used. Geographic Information System (GIS) shapefiles delineating freshwater pearl mussel catchment boundaries were obtained from Ireland’s National Parks & Wildlife Service (National Parks & Wildlife Service, 2024). GIS shapefiles for the first series Ordnance Survey of Ireland and the high-resolution National Land Cover (NLC) Map of Ireland (Tailte Éireann, 2023) were obtained from Tailte Éireann for the sample catchments; the rationale for choosing NLC mapping was that it provided higher-resolution land cover detail in comparison to other data sources that are available for Ireland.

**Table 2:** Map data source, scales and minimum mapping units

Source dataset	Date	Working scale	Minimum mapping units (MMU)
OSI first series mapping 6-inch map (black & white)	Surveyed (Tailte Éireann, 2024): 1829–1834 Published: 1829–1941	1:10,560	6-inch map
National Land Cover Map 2023	2023 National Land Cover Map	-	0.01 −0.05 ha—areal 50 m—linear
Irish Peat Soil Map	2024	-	1 ha
NPWS GIS shapefiles for FPM catchment boundaries	2024	-	-

### 2023 National land cover map

NLC data was published in 2023 and was originally produced by combining data from the OSI photography campaign of 2018, satellite imagery from the European Space Agency (ESA) Sentinel 2 programme and the OSI PRIME 2 vector spatial database. Using remote sensing techniques, NLC determined 36 highly detailed and validated landcover types within Ireland. These were based on 10 million features and produced using a combination of rule-based and machine-learning classification approaches.

The 2023 NLC Map has minimum mapping units of 0.1 ha and 100 m for areal and linear features, respectively (Lydon et al., 2018). The classification thematic accuracy (FI scores) for the dataset is 89% and 79% for level 1 and level 2, respectively (Lydon et al., 2018). An assessment of the spatial delineation of land cover polygons confirmed that all classes had a geometric accuracy of > 80%, with the majority > 85% (Lydon et al., 2018). NLC 2023 has been subjected to rigorous external validation, involving up to 54 validators consisting of expert staff from various public, private, and research organisations, that collectively manually assessed and validated over 22,000 stratified random validation samples (Lydon et al., 2018). The NLC land cover mapping was preferred over CORINE data, as it is reportedly over 250 times more detailed (Lydon et al., 2018).

The 2023 landcover classifications for each catchment were quantified using GIS techniques and allowed between-catchment and historical comparisons. Land cover types were assigned to either wetland or non-wetland cover types. Table 3 lists the land cover classification types selected as wetland cover types. Areas were calculated using the geodesic function in ArcGIS Pro.

Earth Observation (EO) techniques are limited when identifying converted peatlands and historically drained wetlands (Gilet et al., 2024). NLC described the level 2 Blanket Bog land cover (code: 620) as being ‘intact expanses of Blanket Bog systems’. Blanket Bog landcover was systematically manually reviewed using a 500 m by 500 m grid system (Mäyrä et al., 2023). Any areas deemed as hydrologically impaired or damaged (denoted by straight lines of artificial drainage, signs of active/historical turbary activities, roads, etc.) were buffered and deducted from that land cover type and subsequently reclassified using the new cover type ‘Non-intact Blanket Bog’ (Kuemmerlen et al., 2022); 10- to 20-m buffer zones were utilised to account for the contributing effects of localised hydrological damage, depending on topography, and were applied based on expert opinion. Intact Blanket Bog (net) coverage was determined by deducting the ‘Non-intact Blanket Bog’ proportions.

### Ordnance survey Ireland (first series mapping)

GIS techniques were used to quantify the terrestrial wetland cover within OSI’s first series maps, hereafter referred to as OSI 1834; surveying was completed between 1829 and 1834, with 6-inch black and white mapping printed between 1829 and 1941 (Tailte Éireann, 2024).

Land parcels denoted by the following ‘wetland’ symbology were manually digitized to yield total wetland coverage in each catchment: marsh, rough pasture and cropping rock, oziers (*Salix* spp.), and furze or whins (*Ulex* spp.). Given the limitation of symbology used in Ireland’s first series mapping, further sub-classification of wetlands was not possible.

Tailte Éireann (formally Ordnance Survey Ireland) claims that the first series maps are well-regarded by cartographers (Tailte Éireann, 2024), but we cannot currently assess their historical accuracy. Uncertainty in our method for quantifying wetlands includes the assumption that the first series OSI mapping was thematically and geometrically accurate (O’Hara et al., 2024).

Historical documents from Ordnance Survey Ireland outlined the rules followed when surveying marginal lands (documents refer to ‘bog and uncultivated land’, and ‘morasses’) (Seymour, 1980). An objective for the production of the first series OSI maps was to create the most accurate map of marginal lands possible, but surveyors were permitted to decide the position of boundaries separating cultivated and uncultivated land, at the nearest field boundary in uncertain cases (O’Hara et al., 2024). Moreover, lands ≤ 2 ha could be ‘ignored’ when enclosed by large areas, or could not be accessed easily (Seymour, 1980). These uncertainties may have resulted in over or under-estimates of wetlands in remote mountain regions, but it cannot be confirmed how frequently, or to what extent, the latter rules were applied. The format of the first series maps obtained from Tailte Éireann were GIS shapefiles, georeferenced to Irish Transverse Mercator projection (ESPG: 2157) and were not reprojected. While no notable issues with projections were evident, minor geometric errors may have resulted from the reprojection of the original maps from the Cassini projection to the Irish Transverse Mercator projection (O’Hara et al., 2024). During that reprojection, the Cassini projection utilized a central north–south meridian that can distort features within increasing distance from the meridian, but given the relatively small spatial scale of the subject catchments, this error source is not considered to have been significant; Cassini projections can result in discrepancies of up to 50 ft. across adjoining counties (OSi, 2000).

Boundaries denoting exposed rock areas were not delineated at times, or separated from wetlands, on the first series OS mapping. When quantifying the 1834 wetland cover, areas of exposed rock were determined using NLC 2023 data, cross-checked against 2023 satellite imagery and deducted from wetland totals. Our rationale was that areas of bare rock denoted in the 2023 mapping are natural features where vegetation cannot colonize due to exposure and gradients, and would have been similar in 1834.

Lands not listed as wetlands, but underlain by peat soils, were determined by GIS cross-referencing the current Irish Peat Soil Map (ISPM) (Gilet et al., 2024) and were included in wetland totals. The rationale is that land areas underlain by peat soils in 2024 were similar during the 1829–1834 surveying period.

Manual digitization was preferred over GIS-based machine learning techniques (Hashisaki, 1996; Mäyrä et al., 2023; O’Hara et al., 2024; Ståhl & Weimann, 2022), as it allowed the deduction of finer spatial scaled non-wetland features, such as roads, lanes, buildings, quarries, and waterbodies and small order watercourses.

#### Forestry data

Forestry data for the Republic of Ireland were obtained from the Department of Agriculture, Food and the Marine (*private forests, 2021) and Coillte (**forest inventory area data, 2023); Coillte’s dataset was confirmed as up to date as of the 22nd of June 2023. Both datasets were investigated to determine areas of forestry plantation and land ownership within each catchment. Forest (hereafter referred to as ‘forestry’) coverage datasets were preferred rather than overall ownership, given the historical preference for targeting wetlands for that land use (Connolly, 2018; Flynn et al., 2022); Coillte’s forest inventory and private forestry areas were combined to yield the total forest coverage. The extent of Coillte’s forest inventory, Coillte’s property ownership and state-licensed private forestry on peatland and peat soils was determined separately, based on the 2024 ISPM dataset.

#### Analysis

We classified wetland loss as the net negative change in functional wetland extents between 1834 and 2023 (O’Hara et al., 2024). Historical wetland loss was calculated as the difference between the NLC 2023 wetland cover and the OSI 1834 wetland cover areas. Wetland loss was expressed in proportion to (a) the historical 1834 total wetland area (Eq. 1) and (b) the overall catchment area (Eq. 2).

A weighted scoring system was developed, based on expert opinion and relevant literature, that represented the potential storage contribution from wetlands towards catchment hydrological functioning (Table 3). A total Wetland Cover Index (WLCi) value was produced for each catchment based on the product of the weighted scoring system value and the corresponding land cover proportions.

Hydromusindex (HDi) values were calculated for each catchment, based on the 2023 catchment wetland coverage in proportion to the baseline coverage in 1834 (WLi) (Eq. 3) and the WLCi (Eq. 4); higher HDi values indicate increased damage.

The analysis of peatland and peat soils within forested areas was carried out using GIS techniques. This involved clipping the 2024 ISPM GIS shapefile (Gilet et al., 2024) using the forestry GIS shapefiles obtained from Coillte and DAFM. The output allowed the quantification of Coillte’s forest inventory and ownership, and private forestry coverage (geodesic area), on the following features (Table 8):

- Non-peat.
- Raised Bog.
- Lowland Atlantic Blanket Bog.
- Mountain Blanket Bog.
- Fens.
- Other peat soils (i.e. ≥ 10 cm and ≥ 8.6% Organic Matter content).

### Weighted scoring system

Table 3 demonstrates the weighted scoring system produced for the NLC 2023 wetland classifications that represent potential contributions towards water storage. The scoring system values were defined based on expert opinion and relevant literature (Fossitt, 2000; Holden & Burt, 2003; Joyce et al., 2016; Bourgault et al., 2018; Irish Ramsar Wetlands Committee, 2018; Kuemmerlen et al., 2022; Schut & Westbrook, 2022). Whilst the classification thematic accuracy of NLC level 2 data was 79% (Lydon et al., 2018), site-specific analysis (including remote sensing techniques and on-site hydrogeological surveys) is advised for defining higher-resolution land cover weighted scoring(s), to account for factors that influence wetland storage potential (Bring et al., 2022).

**Table 3:** The weighted scoring system produced for the NLC 2023 wetland classifications

Wetland cover type	Weighting score	Rationale
Blanket Bog	10	Associated with upland flat or gently sloping topography (hereafter referred to as ‘Intact Blanket Bog’). Peat depths vary between 1-7m + Storage capacity depends on various factors, including peat depth/area and gradient The weighted score for Non-intact Blanket Bog was 6, similar to Cutover Bog
Fens	9	Fed by groundwater or moving surface waters. Extremely wet, low-lying areas. Storage capacity depends on scale
Swamp	8	Generally occupy a zone at the transition from open water to terrestrial habitats and are inundated with water throughout the year. Storage capacity depends on scale
Wet Heath	7	Occurs on shallow peat or sandy soils, with impeded drainage. Peat depth varies between 15-50cm +
Bare Peat	6	Storage capacity depends on the remaining peat depth and drainage modifications
Cutover Bog	6	Storage capacity depends on the remaining peat depth and drainage modifications
Wet Grassland	5	Occurs on saturated and/or peaty soils, and shallow wet peats, that are too dry or steep for deep peat accumulation
Dry Heath	2	Associated with dry/free-draining acidic to circumneutral soils. Peat depth ≤ 0.5m

### Hydromusindex (HDi)

The wetland net loss between 1834 (T_0_) and 2023 (T_1_) was expressed in two ways, a percentage of the total wetland cover area in 1834 (Eq. 1) and a percentage of the overall catchment area (Eq. 2). The Wetland Loss Index (WLi) represents the 2023 catchment wetland areal coverage in proportion to the 1834 baseline scenario (Eq. 3).

1 $$\mathrm{Wetland}\;\mathrm{loss}\;(1)=\frac{\mathrm{Wetland}\;\mathrm{loss}\;({\mathrm T}_{0:1834}-{\mathrm T}_{1:2023})}{\mathrm{Total}\;\mathrm{wetland}\;\mathrm{area}\;({\mathrm T}_{0:1834})}\times100\%$$

2 $$\mathrm{Wetland}\;\mathrm{loss}\;(2)=\frac{\mathrm{Wetland}\;\mathrm{loss}\;({\mathrm T}_{0:1834}-{\mathrm T}_{1:2023})}{\mathrm{Catchment}\;\mathrm{area}}\;\times\;100\%$$

3 $$\mathrm{Wetland}\;\mathrm{Loss}\;\mathrm{Index}\;(\mathrm{WLi})=\frac{{\mathrm{Catchment}\;\mathrm{wetland}\;\mathrm{area}}_{\mathrm T1}}{{\mathrm{Historical}\;\mathrm{Catchment}\;\mathrm{wetland}\;\mathrm{area}}_{\mathrm T0}}$$

The Wetland Cover Index (WLCi) value for each catchment was the sum of the products of the catchment proportional coverage of each wetland cover type and its corresponding weighted score (Eq. 4). The Hydromusindex (HDi) value for each catchment was produced using the WLi and the WLCi (Eq. 5). Higher HDi values indicate increased damage.

4 $$WLCi=\Sigma \left(LC1{T}_{1}+LC2{T}_{1\cdots \cdots }{LC}_{n}{T}_{1}\right)$$

5 $$Hydromusindex\left(HDi\right)=1/\left(\frac{\sqrt{{\left(WLi\right)}^{2}+{\left(WLCi\right)}^{2}}}{\sqrt{2}}\right)\times 100$$

## Results and discussion

### 2023 catchments and wetlands

Catchment sizes ranged from 17.45 km^2^ (Glaskeelan) to 146.49 km^2^ (Newport). The number of land cover types within the sample catchments ranged between 22 and 27 and varied depending on catchment morphology, size, and the extent of land use, land use change, and forestry (LULUCF) (Table 4). Three peatland types (Intact Blanket Bog, Bare and Cutover Bog), Fens, Swamps, Wet Heath, Dry Heath, and Wet Grassland were selected as wetland types.

The dominant land cover types within sample catchments were primarily Blanket Bog (range: 7.85 to 55.04%), Wet Heath (range: 0.82 to 29.23%), Wet Grassland (range: 2.17 to 37.52%), Dry Heath (range: 0.03 to 22.41%), and Transitional Forestry (range: 2.71 to 13.56%). ‘Exposed rock and sediment’ coverage was lowest in the Owenriff (0.23%) and highest in the Caragh (4.93%). ‘Lake and Pond’ proportions varied considerably between the catchments listed within the ‘Top 8’ (range: 0.56 to 4.60%) and the remaining catchments (range: 1.77 to 4.53%). ‘Lake and Pond’ proportions in the Owenriff (4.60%) and Glaskeelan (3.48%), two of Ireland’s premier freshwater pearl mussel catchments, were comparable to the Clady (4.53%), Eske (4.28%) and Newport (3.09%) catchments. Dry heath proportions were naturally higher in catchments containing elevated mountain ranges and were greatest in the Clady (22.41%).

High proportions of Wet Grassland were noted in catchments subjected to elevated agricultural intensification. The Owenriff (37.52%), Blackwater (16.23%) and Caragh (14.81%) demonstrated a relatively high proportion of Wet Grassland in comparison to the other catchments, which otherwise ranged from 2.17% (Glaskeelan) to 7.74% (Eske).

The Glaskeelan was the least modified catchment, but has one of the most damaged *Margaritifera* populations in Ireland. This is attributed to the fact that all the catchment forestry is located immediately adjacent to the entire length of the mussel habitat. One of the authors (Dr Evelyn Moorkens) collected oral evidence in the 1990s from a local man, born in 1895, who described an exceptionally dense mussel population up to the first drainage and planting in the early 1970s. This demonstrates that the context of the location of damaged wetlands must be a key consideration for restoration plans.

**Fig. 2:**
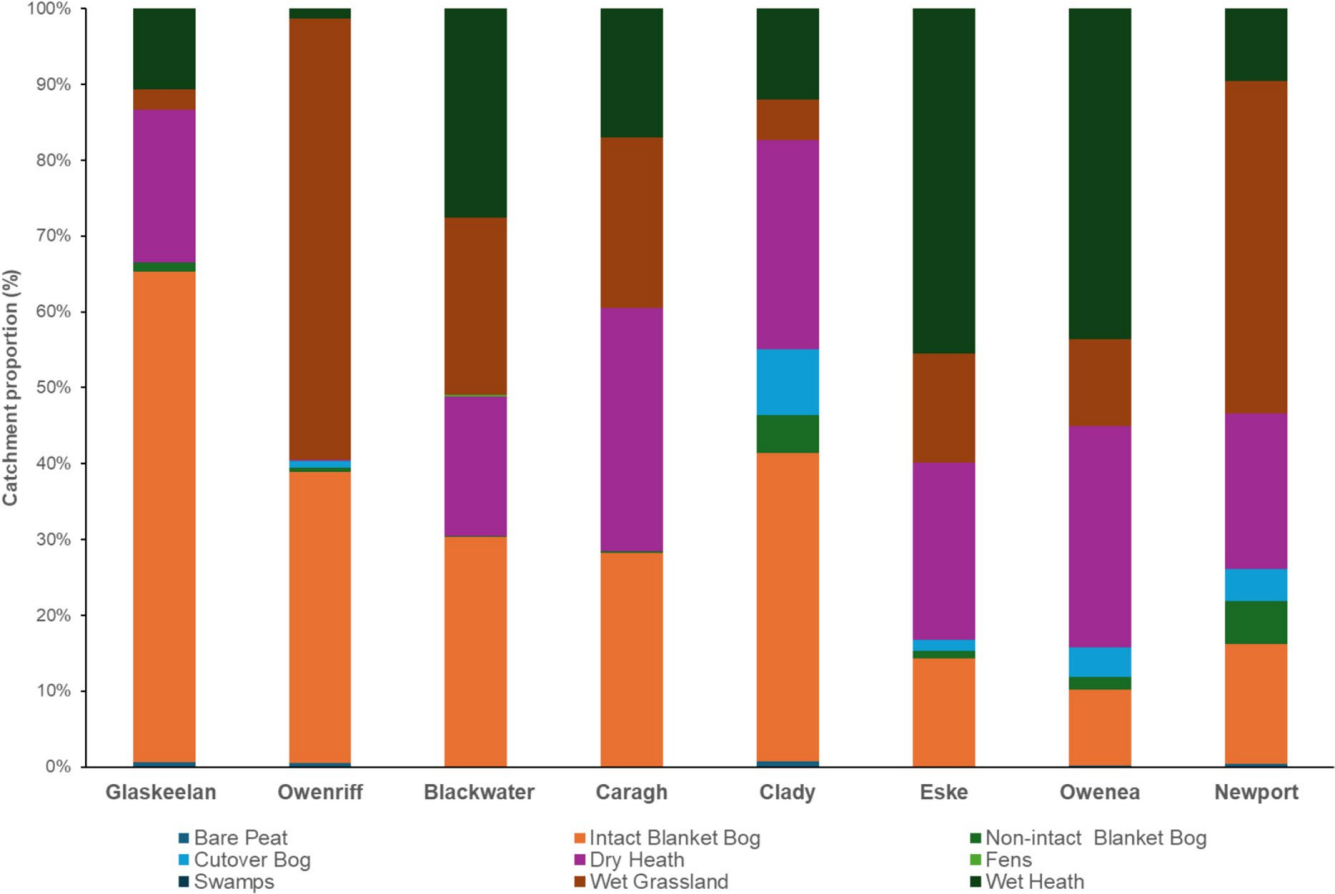
Wetland cover type proportions in the eight sample catchments

Figure 2 highlights the proportions of wetland cover types within the catchments. Catchment proportions of ‘Non-intact Blanket Bog’, representing hydrologically impaired and damaged Blanket Bog, ranged from 0.10% (Blackwater) to 4.12% (Clady); data is not available to determine the original land cover types prior to afforestation. The Newport (3.07%) and Clady (4.12%) contained the highest levels of Non-intact Blanket Bog. Non-intact Blanket Bog in the four less modified catchments averaged 0.40%, less than one-fifth (i.e. 17.9%) of that of the remaining catchments (i.e. 2.21%). The Owenea demonstrated the lowest proportion of Intact Blanket Bog (i.e. 6.76%).

**Table 4:** Land cover types in the studied catchments in absolute (Ha) and relative terms (%) from the 2023 National Land Cover Map of Ireland database (areas of ‘Non-intact’ Blanket 364 Bog indicated in brackets) (Tailte Éireann, 2023)

Catchment	Area	Amenity Grassland	Artificial Waterbodies	Bare Peat	Bare Soil And Disturbed Ground	Blanket Bog*	Bracken	Broadleaved Forest and Woodland	Buildings	Coastal Sediments	Coniferous Forest	Cultivated Land	Cutover Bog	Dry Grassland
**Glaskeelan**	Abs	0.74	0.00	8.48	0	960.6 (17.34)	6.45	11.02	0.25	0.00	20.25	0.00	0.00	0.22
	Prop.(%)	0.04%	0.00%	0.49%	0.00%	55.04%	0.37%	0.63%	0.01%	0.00%	1.16%	0.00%	0.00%	0.01%
**Owenriff**	Abs	47.11	0.00	20.38	0.2	1691.69 (23.66)	5.52	75.55	14.06	0.00	579	0.00	39.13	26.29
	Prop.(%)	0.70%	0.00%	0.30%	0.00%	25.09%	0.08%	1.12%	0.21%	0.00%	8.59%	0.00%	0.58%	0.39%
**Blackwater**	Abs	9.92	0.00	5.06	5.98	1867.72 (8.43)	25.44	184.41	5.54	0.00	743.79	0.00	0.00	59.44
	Prop.(%)	0.11%	0.00%	0.06%	0.07%	21.16%	0.29%	2.09%	0.06%	0.00%	8.43%	0.00%	0.00%	0.67%
**Caragh**	Abs	19.69	0.00	5.16	5.55	2500.36 (19.03)	282.99	234.62	6.5	0.00	370.77	0.00	0.00	1170.77
	Prop.(%)	0.15%	0.00%	0.04%	0.04%	18.71%	2.12%	1.76%	0.05%	0.00%	2.77%	0.00%	0.00%	8.76%
**Clady**	Abs	28.52	3.9	53.66	0.69	3297.72 (366.43)	35.89	37.4	10.34	0.0.	105.77	0.00	624.21	15.47
	Prop.(%)	0.32%	0.04%	0.60%	0.01%	37.04%	0.40%	0.42%	0.12%	0.00%	1.19%	0.00%	7.01%	0.17%
**Eske**	Abs	91.11	0.01	0.00	1.45	942.13 (63.57)	125.89	548.66	37.04	0.00	305.32	1.05	83.04	9.18
	Prop.(%)	0.80%	0.00%	0.00%	0.01%	8.27%	1.11%	4.82%	0.33%	0.00%	2.68%	0.01%	0.73%	0.08%
**Owenea**	>Abs	55.92	0.00	11.28	0.05	990.11 (137.57)	46.05	167.6	22.42	0.00	853.09	0.00	330.99	6.06
	Prop.(%)	0.44%	0.00%	0.09%	0.00%	7.85%	0.37%	1.33%	0.18%	0.00%	6.77%	0.00%	2.63%	0.05%
**Newport**	Abs	41.13	0.00	32.68	17.5	1714.36 (449.74)	147.64	329.32	12.31	0.00	1438.61	6.08	338.9	91.46
	Prop.(%)	0.28%	0.00%	0.22%	0.12%	11.70%	1.01%	2.25%	0.08%	0.00%	9.82%	0.04%	2.31%	0.62%

Catchment	Dry Heath	Exposed Rock and Sediments	Fens	Hedgerows	Improved Grassland	Lakes and Ponds	Marine Water	Mixed Forest	Mudflats	Other Artificial Surfaces	Rivers and Streams	Saltmarsh	Scrub	Swamp
**Glaskeelan**	294.64	50.09	0.00	1.09	22.36	60.75	0.00	1.55	0.00	0.58	4.53	0.00	14.42	0.00
	16.88%	2.87%	0.00%	0.06%	1.28%	3.48%	0.00%	0.09%	0.00%	0.03%	0.26%	0.00%	0.83%	0.00%
**Owenriff**	2.23	15.19	0.00	50.11	380.94	310.32	0.00	6.39	0.00	43.66	32.15	0.00	19.87	0.00
	0.03%	0.23%	0.00%	0.74%	5.65%	4.60%	0.00%	0.09%	0.00%	0.65%	0.48%	0.00%	0.29%	0.00%
**Blackwater**	1128.48	228.19	14.35	79.32	349.52	49.33	0.01	16.51	0.00	17.57	53.7	0.00	124.88	0.00
	12.78%	2.59%	0.16%	0.90%	3.96%	0.56%	0.00%	0.19%	0.00%	0.20%	0.61%	0.00%	1.41%	0.00%
**Caragh**	2827.26	658.12	0.00	80.31	588.8	195.89	0.00	119.62	0.00	18.18	94.89	0.00	97.19	0.00
	21.16%	4.93%	0.00%	0.60%	4.41%	1.47%	0.00%	0.90%	0.00%	0.14%	0.71%	0.00%	0.73%	0.00%
**Clady**	1995.4	404.27	0.00	10.8	83.76	403.13	0.00	2.47	0.00	44.02	28.45	0.21	172.23	0.00
	22.41%	4.54%	0.00%	0.12%	0.94%	4.53%	0.00%	0.03%	0.00%	0.49%	0.32%	0.00%	1.93%	0.00%
**Eske**	1434.63	471.53	0.00	197.85	1927.64	487.53	0.00	57.5	0.00	110.73	55.28	0.00	145.64	0.00
	12.60%	4.14%	0.00%	1.74%	16.93%	4.28%	0.00%	0.50%	0.00%	0.97%	0.49%	0.00%	1.28%	0.00%
**Owenea**	2462.78	33.62	0.00	36.55	956.4	223.37	0.00	5.07	0.09	75.35	63.74	0.00	136.51	2.85
	19.53%	0.27%	0.00%	0.29%	7.59%	1.77%	0.00%	0.04%	0.00%	0.60%	0.51%	0.00%	1.08%	0.02%
**Newport**	1631.65	89.58	0.00	171.67	1403.31	452.62	0.17	31.59	0.00	43.02	75.42	0.00	149.9	0.6
	11.14%	0.61%	0.00%	1.17%	9.58%	3.09%	0.00%	0.22%	0.00%	0.29%	0.51%	0.00%	1.02%	0.00%

Catchment	Transitional Forest	Treelines	Ways	Wet Grassland	Wet Heath									
**Glaskeelan**	89.65	1.41	1.99	37.82	156.27									
	5.14%	0.08%	0.11%	2.17%	8.95%									
**Owenriff**	725.25	28.45	43.27	2529.77	55.08									
	10.76%	0.42%	0.64%	37.52%	0.82%									
**Blackwater**	612.59	45.97	64.22	1440.57	1694.68									
	6.94%	0.52%	0.73%	16.32%	19.20%									
**Caragh**	516.77	24.99	68	1978.95	1496.07									
	3.87%	0.19%	0.51%	14.81%	11.20%									
**Clady**	241.55	6.24	48.08	386.36	863.33									
	2.71%	0.07%	0.54%	4.34%	9.70%									
**Eske**	398.4	139.95	144.02	881.36	2791.76									
	3.50%	1.23%	1.26%	7.74%	24.51%									
**Owenea**	1353.82	31.86	91.62	965.36	3685.18									
	10.74%	0.25%	0.73%	7.66%	29.23%									
**Newport**	1985.77	59.11	120.28	3504.48	760.3									
	13.56%	0.40%	0.82%	23.92%	5.19%

### 1834 wetlands

Table 5 outlines the total wetland cover in 1834 and 2023, wetland loss, and the total ‘forest inventory’ area for each catchment. Based on OSI 1834 data, the percentage wetland coverage within sample catchments ranged between 59.72% (Eske) to 89.85% (Glaskeelan); the average catchment wetland cover was 81.82%.

**Table 5:** Wetland cover, wetland loss (1834–2023) and forestry coverage (2021* & 2023**) within the sample catchments

Catchment	1834 Wetland cover (m^2^) (T_o_)	2023 Wetland cover (m^2^) (T_1_)	Wetland loss (m^2^) (1834–2023) (T_o_-T_1_)	Wetland loss (% of 1834) (T_o_-T_1_)	Catchment wetland coverage (1834) (T_o_)	Catchment wetland coverage (2023) (T_1_)	Wetland loss (% catchment) (2023–1834) (T_o_-T_1_)	Total forestry coverage (m^2^)	Forestry coverage (% catchment)	Forestry & wetland loss difference (1834–2023)
Glaskeelan	15,681,134	14,578,157	1,102,977	7.03%	89.85%	83.53%	6.32%	1,292,158	7.40%	1.08%
Owenriff	56,371,350	43,382,799	12,988,551	23.04%	83.62%	64.35%	19.27%	16,596,813	24.62%	5.35%
Blackwater	74,611,858	61,508,638	13,103,220	17.56%	84.52%	69.68%	14.84%	15,058,183	17.06%	2.21%
Caragh	114,798,130	88,078,064	26,720,066	23.28%	85.92%	65.92%	20.00%	13,047,006	9.76%	−10.23%
Clady	78,381,775	72,206,770	6,175,005	7.88%	88.03%	81.10%	6.94%	5,435,696	6.10%	−0.83%
Eske	68,018,388	61,329,225	6,689,163	9.83%	59.72%	53.85%	5.87%	14,022,114	12.31%	6.44%
Owenea	107,271,820	84,485,573	22,786,247	21.24%	85.08%	67.01%	18.07%	27,407,038	21.74%	3.67%
Newport	113,977,493	79,829,674	34,147,818	29.96%	77.80%	54.49%	23.31%	39,257,642	26.80%	3.49%
Average	78,638,994	63,174,863	15,464,131	17.48%	81.82%	67.49%	14.33%	16,514,581	15.72%	1.40%
Minimum	15,681,134	14,578,157	1,102,977	7.03%	59.72%	53.85%	5.87%	1,292,158	6.10%	−10.23%
Maximum	114,798,130	88,078,064	34,147,818	29.96%	89.85%	83.53%	23.31%	39,257,642	26.80%	6.44%

The 1834 wetland coverage in the four less modified catchments ranged from 83.26% (Owenriff) to 89.85% (Glaskeelan). Historical wetland cover in the Clady (88.03%) and Owenea (85.08%) was comparable to the latter and exceeded that of *all* sites, except for the Glaskeelan. The Eske (59.72%) and Newport (77.80%) had the lowest historical catchment-proportional wetland coverages, but contained relatively large upland lake systems (NLC 2023 Lake and Pond coverage: Eske 4.28%; Newport 3.09%). This suggests the importance of increased storage provided by these waterbodies within such catchments, in terms of hydrological functioning.

### Wetland loss (1834–2023)

**Fig. 3:**
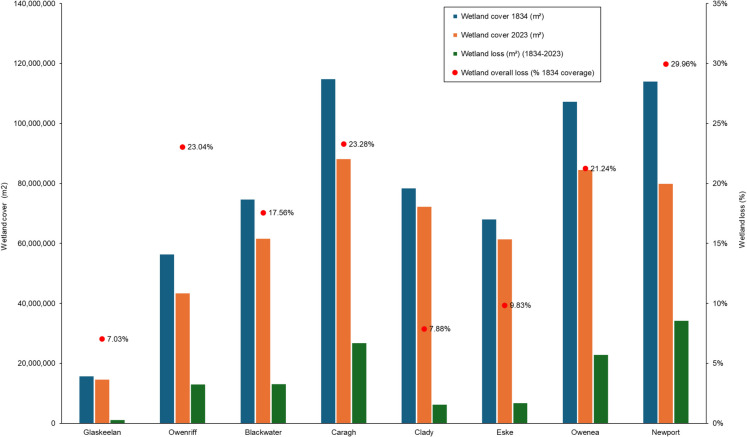
Wetland cover in 1834 and 2023, and wetland loss in proportion to the 1834 baseline

Table 5 outlines the results of wetland proportions between both sample periods, wetland loss and the extent of forestry within the eight sample catchments. Figure 3 illustrates wetland coverage and wetland loss in contrast to 1834 coverage.

The percentages of wetland loss between 1834 and 2023 ranged between 7.03% (Glaskeelan) and 29.96% (Newport), the latter accounting for 23.31% of the overall catchment area. Considerable wetland loss was evident at the Caragh (23.28%), Owenriff (23.04%), and Owenea (21.24%), accounting for 20.00%, 19.27%, and 18.07% of total catchment areas, respectively.

The percentage of wetland loss was relatively low in the Glaskeelan (7.03%), Clady (7.88%), and the Eske (9.83%), equalling 6.32%, 6.94%, and 5.87% of each catchment, respectively. Wetland loss at the Blackwater (17.56%) was marginally less than the 17.48% catchment average.

### Hydromusindex (HDi)

**Fig. 4:**
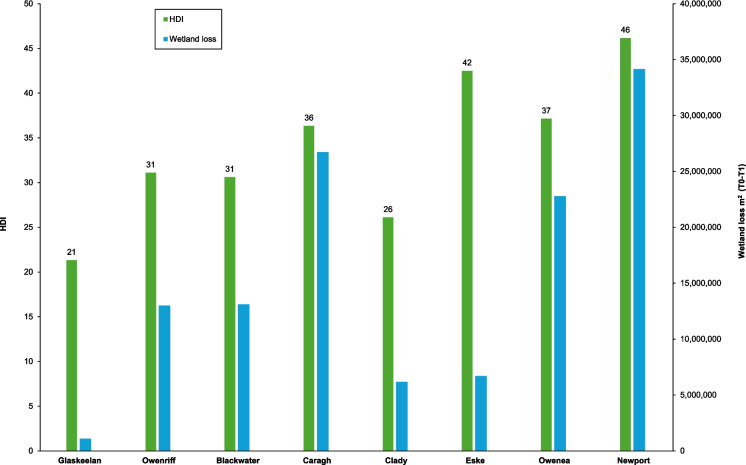
Wetland loss between 1834 and 2023, and catchment Hydromusindex (HDi) values

HDi values were produced for the eight sample catchments (Table 7 and Fig. 4), with higher values indicating increased damage. The Wetland Cover Index (WLCi) was greatest for the Glaskeelan catchment (i.e. 6.57) (Table 6); WLCi values were produced for all sample catchments in this manner. The catchment-proportional wetland cover ratios (i.e. LC ratios) produced for the Glaskeelan may be used as measurable restoration targets; the original land cover types underlying forested areas (afforestation was undertaken between 1976 and 2001) in the Glaskeelan remain unknown and accounted for 7.40% of the catchment (Table 5). A Wetland Cover Index (WLCi) representing the historical unmodified baseline can be produced for each catchment by combining all Blanket Bog cover data (Intact Blanket Bog LC1 and Non-Intact Blanket Bog LC2), and the relevant proportion(s) of Cutover Bog LC7.

**Table 6:** The Wetland Cover Index (WLCi) for the Glaskeelan catchment

Wetland cover	Area (Ha)	LC ratios	Weighting score	LC score
Intact Blanket Bog LC1	943.26	0.540	10	5.40
Non-Intact Blanket Bog LC2	17.34	0.010	6	0.06
Fens LC3	0.00	0.000	9	0.00
Swamp LC4	0.00	0.000	8	0.00
Wet Heath LC5	156.27	0.090	7	0.63
Bare Peat LC6	8.48	0.005	6	0.03
Cutover Bog LC7	0.00	0.000	6	0.00
Wet Grassland LC8	37.82	0.022	5	0.11
Dry Heath LC9	294.64	0.169	2	0.34
	**Wetland Cover Index (WLCi)**			**6.57**

**Table 7:** Hydromusindex (HDi) for the eight sample catchments

Catchment	% Wetland loss	WLi	WLCi	HDi
Glaskeelan	7	0.93	6.57	21
Clady	8	0.92	5.34	26
Blackwater	18	0.82	4.55	31
Owenriff	23	0.77	4.48	31
Caragh	23	0.77	3.82	36
Owenea	21	0.79	3.73	37
Eske	10	0.90	3.20	42
Newport	30	0.70	2.98	46

The Wetland Loss Index (WLi) is the proportion of the 2023 wetland cover (m^2^) to that of 1834. WLi values ranged between 0.93 (Glaskeelan) to 0.70 (Newport) (Table 7). The Wetland Cover Index (WLCi) spanned from 6.57 (Glaskeelan) to 2.98 (Newport) (Fig. 4).

HDi values produced ranged from 21 (Glaskeelan) to 46 (Newport), averaging 34. The Newport (46), Eske (42), Owenea (37) and Caragh (36) exhibited the highest HDi scores, thus confirming considerable historical wetland change and associated potential storage issues that could impact hydrological functioning. Scores for the Owenriff (31), Blackwater (31), and Clady (26) were slightly better than the sample average (34).

The results from Table 7 should be viewed in two ways. The first is to design catchment restoration in order to lower the percentage wetland loss until the relevant population becomes sustainable. The second is to use the HDi to prioritise the restoration that maximises the reduction of the HDi.

In 2010, the Clady catchment was ranked as Ireland’s 10th best freshwater pearl mussel catchment, in terms of population size and recruitment status (Moorkens, 2010). The HDi score for the Clady benefited from its relatively large proportion of remaining Intact Blanket Bog, even though it also had the highest Non-intact Blanket Bog coverage (4.12%) overall. However, the movement of water to the Clady River from its upstream lakes is highly modified by weirs and an artificial bypass regulation channel. Dams, weirs and water abstractions have been shown to have negative effects on *Margaritifera* populations (Curley et al., 2022; Sousa et al., 2020; Tamario et al., 2022).

### Opportunities for ‘forest to bog’ restoration within the sample catchments

Wetland degradation, within the catchment-scaled context, is accounted for by the increasing intensification of land use in Blanket Bog habitats by direct peat extraction, drainage, and sometimes peat removal for agricultural intensification, and through legacy afforestation on previously high-value Blanket Bog habitats. The required lowering of water tables for afforestation purposes was achieved firstly through very intensive drainage (Francis & Taylor, 1989), and, as trees matured, through interception and evapotranspiration by the standing crop of trees (Kuemmerlen et al., 2022; Sarkkola et al., 2010). Much of the legacy forestry is state-owned, thus providing the most straightforward opportunity for projects to rehabilitate open peat habitats.

**Fig. 5:**
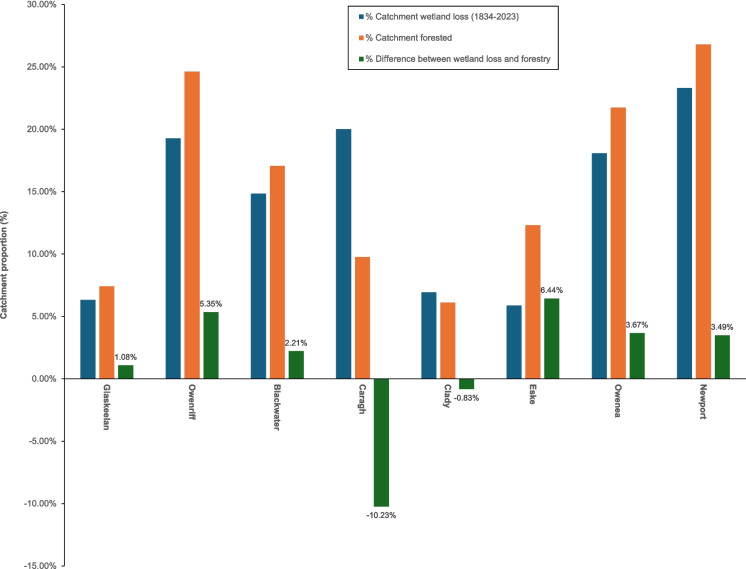
Percentage wetland loss (1834–2023) and forestry within the sample catchments

Coillte forest inventory, Coillte property ownership, and private forestry coverage were compared to the historical wetland loss for each catchment (Table 5 and Fig. 5). Coillte and DAFM’s Forest Service spatial data does not align exactly with the National forest inventory totals; the ownership of ~ 14% of land remains uncertain and differences are attributed mainly to unmapped residential usage (O’Rourke et al., 2023).

The average percentage of forested areas within catchments was 15.72%, with values ranging from 6.10% (Clady) to 26.80% (Newport). The Newport (26.80%) had the greatest proportion of forestry, followed closely by the Owenriff (24.62%) and Owenea (21.74%). The Blackwater (17.06%) and Eske (12.31%) forestry coverage approached the catchment average. In contrast, forestry proportions in the Caragh (9.76%) and Glaskeelan (7.40%) were comparatively lower. The Clady contained the lowest proportion of forestry within all eight catchments (6.10%).

The difference between the percentage of forestry and historical wetland loss (1834–2023) ranged from 6.44% (Eske) to − 10.23% (Caragh). The difference observed at the Clady catchment was marginal (− 0.83%). The percentage of catchment forestry exceeded historical wetland loss in all other catchments, with values ranging from 1.08% (Glaskeelan) to 6.44% (Eske), demonstrating considerable opportunities for wetland restoration. Restoration opportunities were particularly evident at the Owenriff, one of Ireland’s premier freshwater pearl mussel catchments, with forestry coverage (1659.58 Ha/24.62%) exceeding wetland loss by 5.32%. Other notable opportunities for restoration exist in the Blackwater (2.21%), Newport (3.49%), and Owenea (3.67%) catchments.

### National opportunities for ‘forest to bog’ restoration

National-scaled wetland restoration opportunities were investigated based on the extent of (a) Coillte’s ‘forest inventory’, (b) Coillte’s overall land ownership, and (c) private forestry, located on peatlands and peat soils. Datasets used relate to the extent of forestry and land ownership as indicated at the date of receipt; additional afforestation by the date of publication will not significantly alter documented coverage totals, e.g. 2273 Ha was afforested in Ireland in 2022 (Central Statistics Office, 2023).

Based on data provided by Coillte and DAFM, the combined Coillte forest inventory and private forestry coverage on peatlands and peat soils is 338,548.11 Ha, accounting for 41.06% of Ireland’s forestry (Table 8), i.e. 824,534.72 Ha. Coillte property and private forestry on peat soils totalled 342,431.79 Ha (41.53% of Ireland’s forestry) (Table 8).

**Table 8:** Coillte forest inventory, Coillte property and private forestry, on peatlands and peat soils in Ireland

Description	Coillte forest inventory (m^2^)	Coillte property (m^2^)	Private forestry (m^2^)
Non-peat	2,176,213,387	2,308,838,329	2,512,190,961
Raised bog	233,740,600	241,117,575	272,788,005
Lowland Atlantic Blanket Bog	389,784,610	391,696,465	259,810,982
Mountain Blanket Bog	670,992,650	677,965,532	234,381,943
Fens	3,428,791	3,429,248	4,550,312
Other peat soils	712,125,703	734,700,336	603,877,544
Total coverage	4,186,285,741	4,357,747,487	3,887,599,748
Coverage on peatlands and peat soil	2,010,072,354 (201,007.24 Ha)	2,048,909,158 (204,890.92 Ha)	1,375,408,787 (137,540.88 Ha)

### Catchment-scaled restoration

Wetlands are viewed as a globally important natural capital and offer critical ecosystem service capacity. Their restoration requires an integrated understanding of surface and subsurface hydrological and hydrogeological processes (Flynn et al., 2022).

The HDi results indicated considerable damage in terms of historical wetland loss, in seven out of eight sample catchments, with values ranging from 21 at the Glaskeelan to 46 at the Newport. The HDi values for three of Ireland’s ‘Top 8’ catchments, the Caragh, Owenriff and Blackwater, highlighted elevated damage. Blanket Bog and Wet Heath were the dominant wetland cover types contributing towards hydrological storage within the sample catchments.

Within a Norwegian-based study, Gosselin et al. (2023) confirmed that 80% of the *M. margaritifera* population was located in areas having between 1.4 and 18.0% wetland cover only; wetland cover ranged from 0 to 60% in those study sites. In contrast, our study confirmed that wetland cover varied between 53.85 and 83.53%. Furthermore, the majority of *Margaritifera* populations that were previously deemed to be strongholds were located in catchments having wetland cover between 64.35 and 83.83%. We attribute these differences to hydrological resilience provided by ice/snow melt in colder regions (Hoess & Geist, 2020) and increased flow-proportional dilution of pollutants (i.e. sediment and nutrient), relatively stable discharges, deeper glaciated lakes that contribute towards sustainable hydrological conditions, and decreased anthropogenic disturbance at higher altitudes (Gosselin et al., 2023). The absence of significant snow in temperate peaty catchments places a clear emphasis on the restoration of Blanket Bog habitat with high water tables.

Article 14 of The Nature Restoration Law (EU 2024/1991), requires that EU Member States quantify the area that needs to be restored to meet the restoration targets set out in Articles 4 and 5, taking into account the condition of the habitat types referred to in Article 4(1) and (4) and the quality and quantity of the habitats of the species referred to in Article 4(7) that are present in the ecosystems covered by Article 2. Article 4(7) of the Nature Restoration Law (EU 2024/1991) specifically requires restoration measures for freshwater habitats of the species listed in Annexes II, IV and V of Directive 92/43/EEC, such as freshwater pearl mussel, whilst improving the quality of such habitats *until* sufficient quality and quantity of those habitats is achieved; adequate restoration of terrestrial habitat condition is essential for restoring catchment hydrological functioning, and the condition of receiving aquatic ecosystems. Wetland restoration efforts should aim to stabilize baseflow conditions, whilst reducing the scale of flood peaks. The restoration of flow is essential for the recovery of sustainable mussel recruitment in Ireland, and equivalent catchments in Western Europe.

Care is advised in establishing catchment-scaled restoration plans and indices in northern regions where hydrological dynamics differ, since frozen waterbodies and wetlands contribute to baseflow in summer due to snow/ice melt (Hoess & Geist, 2020). Existing land use and geo-topographical constraints will undoubtedly limit the restoration of individual wetlands, thereby requiring a mosaic of wetland types to achieve targeted HDi scores. However, high-scoring wetland cover types, representing maximum cost-benefits, should be prioritised. Care should also be taken in southern populations, particularly Spain and Portugal, where mussel habitat is not only dependent on flow, but reliant on gradient and channel sinuosity, and by important areas of seepage leading to groundwater-surface water interactions at the hyporheic zone. This is also the case in the Irish mussel populations, particularly those of the south-east, where soils are mostly mineral in nature and mussel habitat is primarily found in preferential flow areas, driven by channel morphology and gradient conditions. Forestry has preferentially been planted on the limited peat soils in the upper catchment of these rivers.

We associate the dramatic decline of Ireland’s freshwater pearl mussel population with the combined effects of historical wetland loss, state-subsidised agricultural intensification (Farrell et al., 2024), continuous forestry rotation (and associated activities) on wetlands and peat soil(s) that is licensed and subsidised by the Department of Agriculture, Food and the Marine (DAFM) (Eaton et al., 2008), development authorised by National Planning Authorities, and unauthorised development that has not been prosecuted and restored.

Forestry exceeded wetland loss in all sample catchments, except the Clady and Caragh. Of the latter catchments, a hydroelectric plant is located at the Clady, which includes significant flow regulation (Addy et al., 2012), whereas the Caragh catchment had relatively low Blanket Bog and Wet Heath, and high Exposed Rock and Sediment coverage.

In 2024, 41.06% of Ireland’s total national forestry was confirmed as being located on peatlands and peat soil (≥ 10 cm and ≥ 8.6% Organic Matter content). Drainage associated with forestry activities may limit the potential restoration success of peatlands, depending on the scale of degradation (Connolly, 2018; Farrell et al., 2024). Once degraded, successful peatland restoration can prove difficult due to overlapping impacts on the peat body, hydrology and vegetation (Aitova et al., 2023; de Waard et al., 2024). Whilst optimal peatland restoration to pristine Annex I standards may not be possible in many such cases, for near-bed velocity improvements and to restore the freshwater pearl mussel, it is the scale of water storage function that is important, thereby positively contributing towards overall catchment hydrological functioning. Site-specific assessment, including evaluation of potential wetland restoration efficiency, is advised prior to the deployment of limited resources (Mackin et al., 2024).

Whilst the current study focused on the effects of historical wetland change, we stress the importance of evaluating both the effects on *Margaritifera* and their habitat, resulting from individual catchment-specific stressors, and the combined effects associated with in-combination and accumulative multiple overlapping stressors. We suggest that the latter should focus on land management practices, agricultural intensification, development, flow regulation associated with hydroelectric schemes, hydromorphological alteration relating to fisheries projects and, in particular, the proximity of forestry activities to sensitive *Margaritifera* habitat. As blanket bog restoration opportunities vary from site to site, we also emphasise that, at the stage of choosing within-catchment rehabilitation sites, more detailed peat condition surveys should be undertaken or, where available, data interrogated from other studies as part of the development of site choices and rehabilitation methods (Anderson et al., 2017; Shepherd et al., 2013).

Stream flow is considered the ‘master variable’ and drives riverine ecosystems (Bruland et al., 2003; Poff & Zimmerman, 2010; Poff et al., 1997), and is measurable at the habitat level of *Margaritifera* through near-bed velocity (Moorkens & Killeen, 2014). Until optimum hydrological and baseflow conditions are restored in freshwater pearl mussel catchments, the long-term success of restoration projects will remain elusive. We stress the importance of catchment-scaled restoration projects, whilst there is still time.

### Future work

When using the HDi system, we advocate the use of baseline historical mapping dating to the period immediately before observed declines in *Margaritifera* populations, and refining the wetland scoring system to include a high-resolution assessment of wetland cover condition(s). This should include assessment of wetland hydrological condition(s) using remote sensing techniques (Kalacska et al., 2018; Kuemmerlen et al., 2022; Lees et al., 2018), an evaluation of historical modification to wetlands based on the use of high-resolution LiDAR data (Higginbottom et al., 2018), and modelling of hydrological processes and potential catchment storage capacity (Mackin et al., 2024). In particular, it is important to quantify the modified drainage patterns in each catchment that lead to excessively high flows during high rainfall and extremely low flows during prolonged dry periods. The mapping of functional flood plains is also valuable, as these can ameliorate aggressive high flows. The size and function of upper catchment lakes and other important components, such as seepage zones and other groundwater movement that reaches the hyporheic zone, should be included. Whichever data type(s) and resolution are chosen by end users, the final HDi calibrated system must be consistent within selected sample catchments, to ensure meaningful comparisons.

Wetland restoration projects, aimed at developing sustainable baseflow conditions in any Freshwater Mussel catchment, should be subjected to hydrological modelling to determine the likelihood of success. Continued efforts at restoration should occur if initial restoration projects have been shown to be insufficient. The achievement of adequate near-bed velocity and the improvement of proportions of juvenile mussel survival are the best measures of success, and efforts should continue in an iterative process until sustainability is achieved. This is the process favoured in the *Margaritifera* CEN standard (NSAI, 2017), which also emphasises the importance of regular and appropriate monitoring at the scale of the mussels and their habitat.

In regions of the species range where different hydrological drivers occur, care should be taken that restoration techniques are appropriate for the protection of the maximum habitat area where mussels should be able to thrive.

The HDi method can be expanded to include quantitative and qualitative data suitable for evaluating the combined effects caused by land use, land use change and forestry (LULUCF) based stressors. Further assessment of the locations of the most impactful stressors relative to their effects on key mussel habitat areas can be used to set priorities for sequential conservation actions. This iterative process should not be used as a reason to delay conservation action, which has never been more urgent. However, it highlights the importance of having a regularly updated individual Catchment Management Plan for each *Margaritifera* population, as highlighted by IUCN and as legally required in Irish national legislation to fulfil Articles 2 and 6 of the Habitats Directive (IUCN, in press; NPWS, 2010).

Given the documented rapid decline of Ireland’s *Margaritifera* populations within recent decades, and the urgency for adequate intervention to prevent widespread extinction, we advocate the development and application of a Total Damage Index (TDi), based on the HDi method. The (TDi) should be modelled to allow evaluation of catchment-specific stressors on *Margaritifera* populations (or for application to other targeted protected species and/or habitats), both individually and in combination. We advise, within the next three years, the deployment of minimum 1-m resolution LiDAR data to allow mapping of drainage channels in both the Newport catchment, estimated at €60,000 from EU funds, and all other *Margaritifera* catchments in Ireland. The TDi would allow desktop evaluation of restoration scenarios and a method for defining resource application, by quantitatively determining the decrease in TDi scores attributable to defined stressors. Resources can then be applied based on this data-based technique, in order to maximize restoration efficiency and bolster support for future similar projects: a requirement of the Nature Restoration Law.

## Conclusions

The successful development of the HDi confirmed considerable damage due to historical wetland loss in seven of the eight sample catchments. Results stress the requirement for urgent catchment-scaled intervention to prevent forecasted extinction.

The path to measurable data-based targets has been produced for application in catchment-scaled restoration project(s). However, land use constraints may limit the application of wetland restoration options. In such cases, catchment-scaled restoration evaluation and design should be applied based on a spatial mosaic system, aimed at maximising results. It is advised to continue to rank freshwater pearl mussel catchments in terms of restoration potential and priority, so that financial resources can be deployed in an evidence-based manner to maximise cost–benefit outcomes.

Six catchments demonstrated forestry cover that exceeded historical wetland loss, highlighting opportunities under the EU Nature Restoration Law to restore both Annexed wetland habitats and *Margaritifera* populations.

## Data Availability

No datasets were generated or analysed during the current study. The 2024 Irish Peat Soil Map database was provided courtesy of Dr Louis Gilet (Trinity College Dublin). Boundaries and forest inventory details of private (2021) and semi-state forest plots (2023) were provided by the Department of Agriculture, Food and the Marine (private ownership) and Coillte (semi-state ownership), respectively.

## Abbreviations

*BB*: Blanket Bog
*Blackwater*: Kerry Blackwater River, Co. Kerry
*LC*: Land Cover
*OSI*: Ordnance Survey Ireland
*NLC 2023*: National Land Cover Map
*Ref*: Reference value
*SAC*: Special Area of Conservation
*T*_0_: 1834
*T*_1_: 2023
*WLi*: Wetland Loss index

## Glossary

Wetland loss: The change in wetland areal extent between 1834 and 2023
